# Supplementary material for: Challenges and possibilities in offering support to family caregivers to persons with dementia of non-European background: A qualitative study
Source: PLoS One. 2026 May 13;21(5):e0344264. doi: 10.1371/journal.pone.0344264 (PMC13170823; doi:10.1371/journal.pone.0344264)
Supplement: S1 Table — (DOCX) [file pone.0344264.s001.docx]

**S1 Table 1.** Interview guide used for social care professionals.

| **Interview Questions** |
| --- |
| 1. The noted reluctance shown by family caregivers from non-European backgrounds to use social, health, and care services.   1. Have you observed this phenomenon in your work? If so, what do you think might be the reasons behind it? 2. Have you been in contact with caregivers from non-European backgrounds? How do you reach out to them? 3. What approaches and channels have you used or experimented with? 4. How can we encourage these caregivers to utilize the services available to them? |
| 2. Experiences in meeting caregivers with non-European backgrounds:   1. Describe your recent interactions with caregivers from non-European backgrounds (e.g., communication, interpreters, etc.). 2. Have you received any training in supporting caregivers from non-European backgrounds? Do you find such training meaningful in your work? 3. What resources and support do you offer to help caregivers of individuals with dementia from non-European backgrounds understand and manage the disease? |
| 3. Diversity among caregivers with non-European backgrounds:   1. What opportunities have you experienced in your work with caregivers from non-European backgrounds? 2. What challenges have you encountered in your work with these caregivers? 3. Is your interaction with caregivers from non-European backgrounds different from your interactions with other caregivers? In what ways? Are there similar support needs? 4. Are there any specific language or cultural factors you need to consider when supporting caregivers from non-European backgrounds? |
| 4. Suggestions based on your experience:   1. How can we encourage more caregivers from non-European backgrounds to:  - Use social, health, and care services? - Improve interactions with this group of caregivers? |
