# Supplementary material for: Challenges and possibilities in offering support to family caregivers to persons with dementia of non-European background: A qualitative study
Source: PLoS One. 2026 May 13;21(5):e0344264. doi: 10.1371/journal.pone.0344264 (PMC13170823; doi:10.1371/journal.pone.0344264)
Supplement: Attachment — Submitted filename: Reviewd Manuscript PONE-D-25-46228.pdf [file pone.0344264.s002.pdf]

Manuscript Number PONE-D-25-46228

General comments:

This study addresses an important research gap in understanding the challenges and possibilities in offering support to non-European family caregivers of persons with dementia. I believe this is significant.

Abstract

Page 10 Line 32, Introducing an abbreviation like "FC" for the first time, it is more appropriate to write out in full before the abbreviation in parentheses. This helps readers understand the meaning and context

Lines 42 – 45 address the summary findings from the study; it would have been beneficial to have major and minor or sub-themes since some of the current (8) themes are interrelated (see the findings section for further suggestions)

Notwithstanding, I suggest the authors highlight the key challenges and possibilities which constitute the main aim of this study.

Introduction

Page 13 lines 88-90, Authors should provide additional context or information about the role of social care professionals and family caregivers in Sweden, including relevant policies, Acts and/or guidelines that shape their responsibilities and support systems. In addition, a description of Sweden's healthcare system will give a better understanding of the findings and their implications

Method

Page 15 lines 147-149, I strongly commend the author's use of the COREQ to report the results as attached.

Was any theoretical framework or model employed in this research? If not, it might be beneficial to consider incorporating one, as frameworks provide structure and guidance for a vigorous and systematic methodology and design that strengthen the research.

Findings

Page 17, lines 195 and 196,

The study's exclusive focus on female social care givers limits the generalizability of findings to the broader population in Sweden, a point acknowledged by the authors in the limitations section. Was there a specific rationale for only including women in the sample? If so, it would be helpful for the authors to clearly state this in their write-up.

Page 17, line 201, Table 1 presents demographic data on social care professionals. Were the ages of these professionals recorded? Age could be an influencing factor, as older professionals might bring more life experience compared to younger ones, have developed coping strategies, and have a better understanding of the challenges.

Additionally, it's worth noting the immigration status of the social care professionals themselves. Were any of them immigrants? This could impact their perspectives, potential challenges and enhance the possibilities in offering support to family caregivers to persons with dementia.

Page 18, pages 203 – 207, eight themes emerged from the analyses, and employing a guiding model or framework could have enhanced the process. Nonetheless, I suggest the authors organise these themes into subthemes, as some appear interconnected. This would improve clarity and effectively highlight the key issues.

Discussions and Conclusion

The discussion sections are well-organised and logically presented. To further enhance the write-up, I suggest that authors highlight the study's contributions and implications.
