# Supplementary material for: Challenges and possibilities in offering support to family caregivers to persons with dementia of non-European background: A qualitative study
Source: PLoS One. 2026 May 13;21(5):e0344264. doi: 10.1371/journal.pone.0344264 (PMC13170823; doi:10.1371/journal.pone.0344264)
Supplement: Attachment — Submitted filename: Response to Reviewers.docx [file pone.0344264.s005.docx]

Dear Editor,

Thank you for considering our manuscript for publication in your journal *PLOS One.* We appreciate the constructive comments provided by both the editor and reviewers #1 and #2, which have contributed to improving the quality of our manuscript. Please find below our detailed responses to the editorial comments as well as those from reviewers #1 and #2. As authors, we have addressed all the points raised to help improve the manuscript. All revisions are clearly marked using track changes.

The authors would also like to alert the editor that minor changes have been made to the manuscript in addition to those addressing the editor’s and reviewers’ comments. These changes include improvements in grammar and updates to information such as definitions and demographic data, supported by references in the “Background” section. All revisions are marked using track changes.

Please note that the references in the text as well as the reference list got revised due to updating of a few references. The page numbers in the “COREQ (COnsolidated criteria for REporting Qualitative research” (see S1 Appendix. COREQ Checklist) have also been adjusted due to the changes in the page numbers as a result of the revisions in the manuscript.

Best regards,

Kirsi Tiitinen Mekhail, corresponding author

**Editor’s comment**

| Comments | Actions taken |
| --- | --- |
| **Editor’s comment #1:**  1. Please ensure that your manuscript meets PLOS ONE's style requirements, including those for file naming. The PLOS ONE style templates can be found at  <https://journals.plos.org/plosone/s/file?id=wjVg/PLOSOne_formatting_sample_main_body.pdf> and  [https://journals.plos.org/plosone/s/file?id=ba62/PLOSOne_formatting_sample_title_authors_affiliations.pdf](https://track.editorialmanager.com/CL0/https:%2F%2Fjournals.plos.org%2Fplosone%2Fs%2Ffile%3Fid=ba62%2FPLOSOne_formatting_sample_title_authors_affiliations.pdf/1/010f019a1942abd0-3dc75352-45d2-43fe-a03c-2ec6dd65388a-000000/QQLQDhL4yPMYTt286k3CpwgiBSarShG15TwGdV6tMqk=234) | We thank the editor for this information. We have followed PLOS ONE’s requirements including for file naming. |
| **Editor’s comment #2:**  2. We note that the grant information you provided in the ‘Funding Information’ and ‘Financial Disclosure’ sections do not match.  When you resubmit, please ensure that you provide the correct grant numbers for the awards you received for your study in the ‘Funding Information’ section. | We thank the editor for this comment, the authors have ensured that the grant numbers are correct and presented in the “Funding Information’ section” in the online submission form. |
| **Editor’s comment #3:**  3. Thank you for stating the following financial disclosure: This research received funding from the Swedish Council for Working Life and Social Research (GD-2023/0006).  Please state what role the funders took in the study. If the funders had no role, please state: "The funders had no role in study design, data collection and analysis, decision to publish, or preparation of the manuscript." If this statement is not correct you must amend it as needed. Please include this amended Role of Funder statement in your cover letter; we will change the online submission form on your behalf. | We thank the editor for this comment, the following statement has been included in the cover letter: "The funders had no role in study design, data collection and analysis, decision to publish, or preparation of the manuscript." |
| **Editor’s comment #4:**  4. Thank you for stating the following in the Acknowledgments Section of your manuscript: The authors would like to thank the community-based social care professionals who participated in this study. This research received funding from the Swedish Council for Working Life and Social Research (GD-2023/0006).  We note that you have provided funding information that is not currently declared in your Funding Statement. However, funding information should not appear in the Acknowledgments section or other areas of your manuscript. We will only publish funding information present in the Funding Statement section of the online submission form.  Please remove any funding-related text from the manuscript and let us know how you would like to update your Funding Statement. Currently, your Funding Statement reads as follows: This research received funding from the Swedish Council for Working Life and Social Research (GD-2023/0006).  Please include your amended statements within your cover letter; we will change the online submission form on your behalf. | We thank the editor for this comment. Our funding statement has been removed from the acknowledgement section in the manuscript.  The following statement has been included in the cover letter: "ZNK (Principal Investigator of the project) received funding for this research from the Swedish Council for Working Life and Social Research grant number (GD-2023/0006). Funder website: <https://forte.se/en>.” |
| **Editor’s comment #5:**  5. In this instance it seems there may be acceptable restrictions in place that prevent the public sharing of your minimal data. However, in line with our goal of ensuring long-term data availability to all interested researchers, PLOS’ Data Policy states that authors cannot be the sole named individuals responsible for ensuring data access (<http://journals.plos.org/plosone/s/data-availability#loc-acceptable-data-sharing-methods>).  Data requests to a non-author institutional point of contact, such as a data access or ethics committee, helps guarantee long term stability and availability of data. Providing interested researchers with a durable point of contact ensures data will be accessible even if an author changes email addresses, institutions, or becomes unavailable to answer requests.  Before we proceed with your manuscript, please also provide non-author contact information (phone/email/hyperlink) for a data access committee, ethics committee, or other institutional body to which data requests may be sent. If no institutional body is available to respond to requests for your minimal data, please consider if there any institutional representatives who did not collaborate in the study, and are not listed as authors on the manuscript, who would be able to hold the data and respond to external requests for data access? If so, please provide their contact information (i.e., email address). Please also provide details on how you will ensure persistent or long-term data storage and availability. | Data protection officer can be reached at Karolinska Institutet via email at [dataskyddsombud@ki.se](mailto:dataskyddsombud@ki.se) or by phone at +46 8-524 864 73. |
| **Editor’s comment #6:**  6. When completing the data availability statement of the submission form, you indicated that you will make your data available on acceptance. We strongly recommend all authors decide on a data sharing plan before acceptance, as the process can be lengthy and hold up publication timelines. Please note that, though access restrictions are acceptable now, your entire data will need to be made freely accessible if your manuscript is accepted for publication. This policy applies to all data except where public deposition would breach compliance with the protocol approved by your research ethics board. If you are unable to adhere to our open data policy, please kindly revise your statement to explain your reasoning and we will seek the editor's input on an exemption. Please be assured that, once you have provided your new statement, the assessment of your exemption will not hold up the peer review process. | The datasets generated and/or analysed in the current study are not publicly available due to the ethical approval (registration number Dnr: 2023-02911-01), which states that the use of interview data should respect the anonymity and integrity of participants, only be accessed as a whole by the research group, and be stored locked with passwords. The corresponding author can be contacted for further information, and the datasets of the current study can be made available from Principal Investigator of the study Zarina Nahar Kabir upon reasonable request. |
| **Editor’s comment #7:**  7. Please amend the manuscript submission data (via Edit Submission) to include author Zarina Nahar Kabir and Marie Tyrrell | The manuscript submission data (via Edit Submission) has been revised to include authors Zarina Nahar Kabir and Marie Tyrrell. |
| **Editor’s comment #8:**  8. Please amend your authorship list in your manuscript file to include author Zarina Nahr Kabir and Marie Tyrell | The authorship list in the manuscript has been revised to include authors Zarina Nahar Kabir and Marie Tyrrell. |
| **Editor’s comment #9:**  9. Please include captions for your Supporting Information files at the end of your manuscript, and update any in-text citations to match accordingly. Please see our Supporting Information guidelines for more information: <http://journals.plos.org/plosone/s/supporting-information>. | Captions for our Supporting Information files have been added, including updates of any in-text citations to match the names of the files accordingly. |
| **Editor’s comment #10:**  10. Please remove all personal information, ensure that the data shared are in accordance with participant consent, and re-upload a fully anonymized data set.  Note: spreadsheet columns with personal information must be removed and not hidden as all hidden columns will appear in the published file.  Additional guidance on preparing raw data for publication can be found in our Data Policy (<https://journals.plos.org/plosone/s/data-availability#loc-human-research-participant-data-and-other-sensitive-data>) and in the following article: <http://www.bmj.com/content/340/bmj.c181.long>. | We are not sure what this comment is referring to since we have not uploaded any data and thus no personal information was shown either. |
| **Editor’s comment #11:**  11. If the reviewer comments include a recommendation to cite specific previously published works, please review and evaluate these publications to determine whether they are relevant and should be cited. There is no requirement to cite these works unless the editor has indicated otherwise. | We thank the editor for providing this information but neither of the reviewers have suggested any specific publication for reference. |

**Reviewer 1’s comments**

| Comments | Actions taken |
| --- | --- |
| **Reviewer #1’s comment #1:** Title: The current title is rather long and somewhat dense, which may make it confusing to readers. I recommend revising it for clarity and conciseness while still capturing the essence of the study. | We thank the reviewer for this suggestion. The title has been revised for clarity and conciseness to: “Challenges and possibilities in offering support to family caregivers to persons with dementia of non-European background: A qualitative study” |
| **Reviewer #1’s comment #2:** Abstract: The opening sentence is unclear and could be restructured for better readability and impact. A more precise formulation would strengthen the abstract. | We thank the reviewer for the suggestion. The opening sentence has been revised and restructured in the abstract. |
| **Reviewer #1’s comment #3:**  Participants: More detail should be provided about the study participants. For instance, elaborating on their expected job roles and levels of experience would help give readers a clearer understanding of who was included in the study and the context of their perspectives. | We thank the reviewer for this comment. A clarification of the nature of social care professionals' role and work has been added in the “Introduction section” with supporting references (20-25). |
| **Reviewer #1’s comment #4:**  Introduction: The introduction is well written and provides a solid foundation for the study. | We thank the reviewer for this feedback. |
| **Reviewer # 1’s comment #5:** Discussion: This section would benefit from more in-depth analysis of the identified themes. Expanding on the findings and critically engaging with them in light of existing literature would add significant value. Additionally, the discussion should explicitly outline recommendations for future research based on the study’s findings. | We thank the reviewer for this comment. In this manuscript, our primary aim was to highlight the key findings that emerged from the data, with a focus on the perspectives of social care professionals. While we acknowledge the value of further theoretical expansion, we have chosen to maintain a concise discussion to preserve the clarity and focus of the study. We believe the current discussion sufficiently reflects the core insights and practical implications of the findings. However, we have ensured that the key themes are clearly presented and linked to relevant literature, and we have added a brief section on implications of our study including suggestion or future research to address this point. |
| **Reviewer #1’s comment #6:** Limitations: The limitation that all participants were women should be further developed. Please explain in more detail how this may have influenced the findings and whether this reflects the broader demographics of the profession. | We thank the reviewer for this comment. As stated in the limitations, the fact that all participants were women reflects the gender distribution among social care professionals in Sweden, as supported by references (20,26). However, the authors have now elaborated on how gender preference among FCs when receiving support may influence the uptake of available support services. This is particularly relevant given that most FCs in Sweden are women (supporting reference: 47). |
| **Reviewer #1’s comment #7:**  Participants Background: Consider adding a paragraph about the nature of social care professionals’ work, including how they are typically recruited and chosen. This background is important, as it may be directly related to the challenges and possibilities they identified. | Please see response for “Reviewer #1’s comment #3”. |

**Reviewer #2’s comment**

| Comments | Actions taken |
| --- | --- |
| **Reviewer #2’s comment #1:**  Reviewer #2: General comments: This study addresses an important research gap in understanding the challenges and possibilities in offering support to non-European family caregivers of persons with dementia. I believe this is significant. | We thank the reviewer for this comment regarding our study. |
| **Abstract**  **Reviewer #2’s comment #2:**  **Page 10 Line 32,** Introducing an abbreviation like "FC" for the first time, it is more appropriate to write out in full before the abbreviation in parentheses. This helps readers understand the meaning and context. | We thank the reviewer for this comment. Family caregivers, including the abbreviation (FCs) have been written out the first time it is mentioned in the abstract. |
| **Reviewer #2’s comment #3:**  **Lines 42 – 45** address the summary findings from the study; it would have been beneficial to have major and minor or sub-themes since some of the current (8) themes are interrelated (see the findings section for further suggestions)  Notwithstanding, I suggest the authors highlight the key challenges and possibilities which constitute the main aim of this study. | We thank the reviewer for this insightful suggestion regarding the addition of subthemes. However, the Systematic Text Condensation (STC) method already provides a structured analytical process, beginning with the identification of preliminary themes, as described in the “Data analysis” section. According to Malterud (2012) (reference 35), the method presents data in “themes” rather than “themes and subthemes/major or minor themes”. Furthermore, as discussed by ref: Morse, J.M. (2008) Confusing categories and themes. Qual Health Res. 2008 Jun;18(6):727-8.doi: 10.1177/1049732308314930), themes as opposed to categories can be interconnected and “co-occur” across other themes. Therefore, the authors have discussed the suggestion and decided not to make any changes to the analysis based on the method used. Additionally, the key challenges and possibilities which constitute the main aim of this study have been addressed in the “Discussion section” of the manuscript. |
| **Introduction**  **Reviewer #2’s comment #4:**  **Page 13 lines 88-90**, Authors should provide additional context or information about the role of social care professionals and family caregivers in Sweden, including relevant policies, Acts and/or guidelines that shape their responsibilities and support systems. In addition, a description of Sweden's healthcare system will give a better understanding of the findings and their implications. | We thank the reviewer for this comment. In addition to the corrections made, as noted in our response to “Reviewer 1’s comment #3”, a description of the Swedish care system has been added to the introduction with supporting references (17). |
| **Method**  **Reviewer #2’s comment #5:**  **Page 15 lines 147-149**, I strongly commend the author's use of the COREQ to report the results as attached.  Was any theoretical framework or model employed in this research? If not, it might be beneficial to consider incorporating one, as frameworks provide structure and guidance for a vigorous and systematic methodology and design that strengthen the research. | In conducting interviews with social care professionals about their experiences, we chose not to use a predefined theoretical framework. This decision was based on the exploratory nature of our study. Our aim was to allow the lived experiences of the participants to guide the findings without being constrained or interpreted through a particular theoretical lens. By applying this type of approach, we prioritised an openness to emerging themes which may otherwise be overlooked if filtered through a specific theory. We hope the reviewer will find the explanation satisfactory. |
| **Findings**  **Reviewer #2’s comment #6:**  **Page 17, lines 195 and 196,**  The study's exclusive focus on female social care givers limits the generalizability of findings to the broader population in Sweden, a point acknowledged by the authors in the limitations section.  Was there a specific rationale for only including women in the sample? If so, it would be helpful for the authors to clearly state this in their write-up. | We thank the reviewer for this comment. For further clarification regarding the gender distribution among social care professionals in Sweden, please see the response for “Reviewer 1’s comment #6”. |
| **Reviewer #2’s comment #7:**  **Page 17, line 201, Table 1** presents demographic data on social care professionals. Were the ages of these professionals recorded? Age could be an influencing factor, as older professionals might bring more life experience compared to younger ones, have developed coping strategies, and have a better understanding of the challenges.  Additionally, it's worth noting the immigration status of the social care professionals themselves. Were any of them immigrants? This could impact their perspectives, potential challenges and enhance the possibilities in offering support to family caregivers to persons with dementia. | We thank the reviewer for this insightful comment. The demographic data mentioned in the comment regarding the social care professionals were not recorded in this study. The background information we had on the study participants, in addition to gender, were on their geographical location, educational background and job title which are all reported in Table 1. |
| **Reviewer #2’s, comment #8:**  **Page 18, pages 203 – 207**, eight themes emerged from the analyses, and employing a guiding model or framework could have enhanced the process. Nonetheless, I suggest the authors organise these themes into subthemes, as some appear interconnected. This would improve clarity and effectively highlight the key issues. | Please see the response provided for “Reviewer #2’s, comment #3” and “ Reviewer #2’s, comment #5” |
| **Reviewer #2’s, comment #9:**  **Discussions and Conclusion**  The discussion sections are well-organised and logically presented. To further enhance the write-up, I suggest that authors highlight the study's contributions and implications. | We thank the reviewer for this comment. Implications of this study have been added at the end of the “Discussion” section. |
