# Supplementary material for: Challenges and possibilities in offering support to family caregivers to persons with dementia of non-European background: A qualitative study
Source: PLoS One. 2026 May 13;21(5):e0344264. doi: 10.1371/journal.pone.0344264 (PMC13170823; doi:10.1371/journal.pone.0344264)
Supplement: Attachment — Submitted filename: Review PLOS ONE_2_Challenges and possibilities FC.docx [file pone.0344264.s004.docx]

**"Challenges and possibilities in offering support to family caregivers to persons with dementia of non-European background: A qualitative study"**

I acknowledge and commend the authors for addressing the comments, which undoubtedly enhances the quality of the manuscript and contributes to the peer review process.

However, I noted that the ***Reviewer #2's comment #7*** was not fully addressed, and this omission may have an impact on the study outcome. The authors have acknowledged that the demographic data pertaining to social care professionals, as highlighted in the comment, were not captured in the study. Given the potential constraints in revisiting the field, **I recommend that the authors consider acknowledging this as a limitation of the study.**

**See the main reviewer comment: *(Reviewer #2's comment #7)***

“Page 17, line 201, Table 1 presents demographic data on social care professionals. Were the ages of these professionals recorded? Age could be an influencing factor, as older professionals might bring more life experience compared to younger ones, have developed coping strategies, and have a better understanding of the challenges. Additionally, it's worth noting the immigration status of the social care professionals themselves. Were any of them immigrants? This could impact their perspectives, potential challenges and enhance the possibilities in offering support to family caregivers to persons with dementia.”
