# Supplementary material for: Challenges and possibilities in offering support to family caregivers to persons with dementia of non-European background: A qualitative study
Source: PLoS One. 2026 May 13;21(5):e0344264. doi: 10.1371/journal.pone.0344264 (PMC13170823; doi:10.1371/journal.pone.0344264)
Supplement: Attachment — Submitted filename: Response to reviewers 14th of April 2026.docx [file pone.0344264.s006.docx]

**Dear Editor,**

Thank you for considering our manuscript for publication in *PLOS One* and for providing constructive and valuable comments. We appreciate the time and effort devoted to reviewing our work.

We have carefully addressed all points raised. The revisions are highlighted in the document titled *“Revised Manuscript with Track Changes”* and are also summarized in the accompanying response table.

We hope that these revisions meet the journal’s expectations and enhance the clarity and quality of the manuscript. We remain grateful for the opportunity to revise and resubmit our work.

Sincerely,
**Kirsi Tiitinen Mekhail**
Corresponding author

| **Comments** | **Action taken** |
| --- | --- |
| **Reviewer #2's comment #7**  See the main reviewer comment: (Reviewer #2's comment #7)  “Page 17, line 201, Table 1 presents demographic data on social care professionals. Were the ages of these professionals recorded? Age could be an influencing factor, as older professionals might bring more life experience compared to younger ones, have developed coping strategies, and have a better understanding of the challenges. Additionally, it's worth noting the immigration status of the social care professionals themselves. Were any of them immigrants? This could impact their perspectives, potential challenges and enhance the possibilities in offering support to family caregivers to persons with dementia.” | We thank the editor for drawing attention to Reviewer 2’s comment#7.  In response, we have revised the manuscript by adding description that:  “Demographic data such as participants’ age or immigration status were not collected.” (page 11 line 245).  Additionally, we have added a statement in the limitations section acknowledging the partial collection of demographic data and the potential implications this may have for the interpretation of the findings.  (Page 27 line 626): “However, a limitation of the study is that demographic data for social care professionals were only partially captured, with variables such as age and immigration status omitted, which could have added valuable insight for the analysis. “ |
| **Editor’s comment:**  - Sampling and participant recruitment, specifically: details about the purposive sampling; what were the inclusion criteria; numbers contacted/agreed/refused to participate; how was sample size determined... | We thank the editor for raising this point. We have revised **Study settings and recruitment** section to address the raised comment: (page 8 line 174-)  **Study setting and recruitment**  “Social care professionals were eligible for inclusion if they were employed within municipal social care services in Sweden and provided support to FCs of older adults. Purposive sampling was used to identify suitable municipalities and professionals. Specifically, municipalities were selected based on having a high proportion of residents with immigrant backgrounds, as reported by the Statistics Sweden [34] to ensure relevance to the study’s focus. Contact information for social care professionals (e.g., email addresses and telephone numbers) was retrieved from official municipal websites.  More than 100 email invitations were sent to social care units across the selected municipalities. As, non‑responders did not actively indicate refusal, it was not possible to determine the exact number of individuals who declined to participate. Professionals who responded and expressed willingness were enrolled in the study. To broaden the pool of potential participants, snowball sampling was also employed, whereby participating professionals were invited to refer colleagues who met the inclusion criteria. The final sample size was determined based on the study’s information power...” |
| **Editors comment: -** Duration of interviews | The duration of the interviews has been added (Page 9 line 202-):  “The length of the interviews ranged from 21.02 to 46.32 minutes.” |
| **Editors comment:**  - Who conducted interviews and information about their background/expertise; | We thank the editor for this question. Clarification regarding who conducted the interviews has been added under **Data collection**: (page 9 line 199):  “… conducted primarily by the co–first authors, KTM and ASK, both of whom are registered nurses holding PhD degrees. MT, a registered nurse with a PhD, and ZNK, Associate Professor of Public Health (PhD), also participated in selected interviews...” |
| **Editor’s comment:** - Further clarification on why this particular method (Systematic Text Condensation) was chosen for data analysis over other methods of qualitative data analysis. | We thank the Editor for this comment. We have addressed this point by adding the following sentences under **Data analyses** (page 10 line 214-):  “The approach was further chosen because it provides a transparent, stepwise procedure well aligned with the descriptive aims of the study and allows for systematic cross‑case thematic analysis while remaining close to participants’ accounts. Furthermore, the aim was to produce a structured, practice‑relevant synthesis of professionals’ perspectives.” |
